# Cross-sectional associations between variations in ankle shape by statistical shape modeling, injury history, and race: the Johnston County Osteoarthritis Project

**DOI:** 10.1186/s13047-017-0216-3

**Published:** 2017-07-26

**Authors:** Amanda E. Nelson, Yvonne M. Golightly, Shahmeer Lateef, Jordan B. Renner, Joanne M. Jordan, Richard M. Aspden, Howard Hillstrom, Jennifer S. Gregory

**Affiliations:** 1 Thurston Arthritis Research Center, University of North Carolina at Chapel Hill, 3300 Doc J. Thurston Building, Campus Box 7280, Chapel Hill, NC 27599-7280 USA; 2 University of North Carolina at Chapel Hill School of Medicine, Chapel Hill, NC USA; 3 Department of Epidemiology, Gillings School of Global Public Health, University of North Carolina at Chapel Hill, Chapel Hill, NC USA; 4 Injury Prevention Research Center University of North Carolina at Chapel Hill, Chapel Hill, NC USA; 5 Department of Radiology, University of North Carolina at Chapel Hill, Chapel Hill, NC USA; 6 Department of Orthopaedics, University of North Carolina at Chapel Hill, Chapel Hill, NC USA; 7 Arthritis and Musculoskeletal Medicine, School of Medicine, Medical Sciences and Nutrition, University of Aberdeen, Aberdeen, UK; 8 Leon Root Motion Analysis Laboratory, Hospital for Special Surgery, New York City, NY USA

**Keywords:** Ankle, Injury, Joint shape, Radiography, Racial differences

## Abstract

**Background:**

Injury is an important risk factor for osteoarthritis (OA), a highly prevalent and disabling joint disease. Joint shape is linked to OA, but the interplay of injury and joint shape and their combined role in OA, particularly at the ankle, is not well known. Therefore, we explored cross-sectional associations between ankle shape and injury in a large community-based cohort.

**Methods:**

Ankles without radiographic OA were selected from the current data collection of the Johnston County OA Project. Ankles with self-reported prior injury were included as injury cases (*n* = 108) along with 1:1 randomly selected non-injured ankles. To define ankle shape, a 68 point model on weight-bearing lateral ankle radiographs was entered into a statistical shape model, producing a mean shape and a set of continuous variables (modes) representing variation in that shape. Nineteen modes, explaining 80% of shape variance, were simultaneously included in a logistic regression model with injury status as the dependent variable, adjusted for intra-person correlation, sex, race, body mass index (BMI), baseline OA radiographic grade, and baseline symptoms.

**Results:**

A total of 194 participants (213 ankles) were included; mean age 71 years, BMI 30 kg/m^2^, 67% white and 71% women. Injured ankles were more often symptomatic and from whites. In a model adjusted only for intra-person correlation, associations were seen between injury status and modes 1, 6, 13, and 19. In a fully adjusted model, race strongly affected the estimate for mode 1 (which was no longer statistically significant).

**Conclusions:**

This study showed variations in ankle shape and history of injury as well as with race. These novel findings may indicate a change in ankle morphology following injury, or that ankle morphology predisposes to injury, and suggest that ankle shape is a potentially important factor in the development of ankle OA.

## Background

Injury is a major risk factor for osteoarthritis (OA), a common chronic disease of the joint (cartilage, bone, and synovium) that is leading cause of disability among adults in the United States [1]. Injuries likely accelerate progression to OA in weight bearing joints by altering joint alignment and biomechanics, thereby changing the magnitudes and locations of peak joint forces during movements, resulting in abnormal loading of the cartilage, subchondral bone, and ligamentous structures. Although OA of the ankle [2] is less common than OA of other lower body joints, the etiology is predominantly posttraumatic [2], in contrast to “primary” OA most often seen in the hip and knee. We recently reported an 80% higher odds of ankle OA among those with prior ankle injury [3]. Ankle OA results in substantial decrements in quality of life similar to those seen with severe hip OA [4]. Also notably, the success of joint replacement in ankle OA is markedly less than that at the hip and knee [5], although newer implants and procedures suggest some progress. There is a long latency period of around 20 years, between injury and end-stage ankle OA [6], a time when identification of those at risk could be key in preventing future disability [7].

Sprains and strains, most of which involve the ankle, were the number one cause of Emergency Department evaluation in the U.S. 2010–2013, and are always in the top two [8]. There are many different mechanisms of injury at this complex joint, resulting in a wide range of injury severity from very minor and asymptomatic to severe fractures [9]. However, even minor injuries may affect the biomechanics of the joint, such that an individual could be predisposed to re-injury and chronic instability. Injury could theoretically alter the shape of the joint, resulting in abnormal biomechanics and increased risk for OA. If such alterations in joint shape could be assessed using accessible and affordable conventional radiography, it may be possible to identify individuals at higher risk of developing OA, allowing implementation of strategies (e.g. weight loss, exercise) to reduce that risk. Such alterations may also provide insights into possible biomechanical mechanisms contributing to the development of OA.

**Fig. 1:**
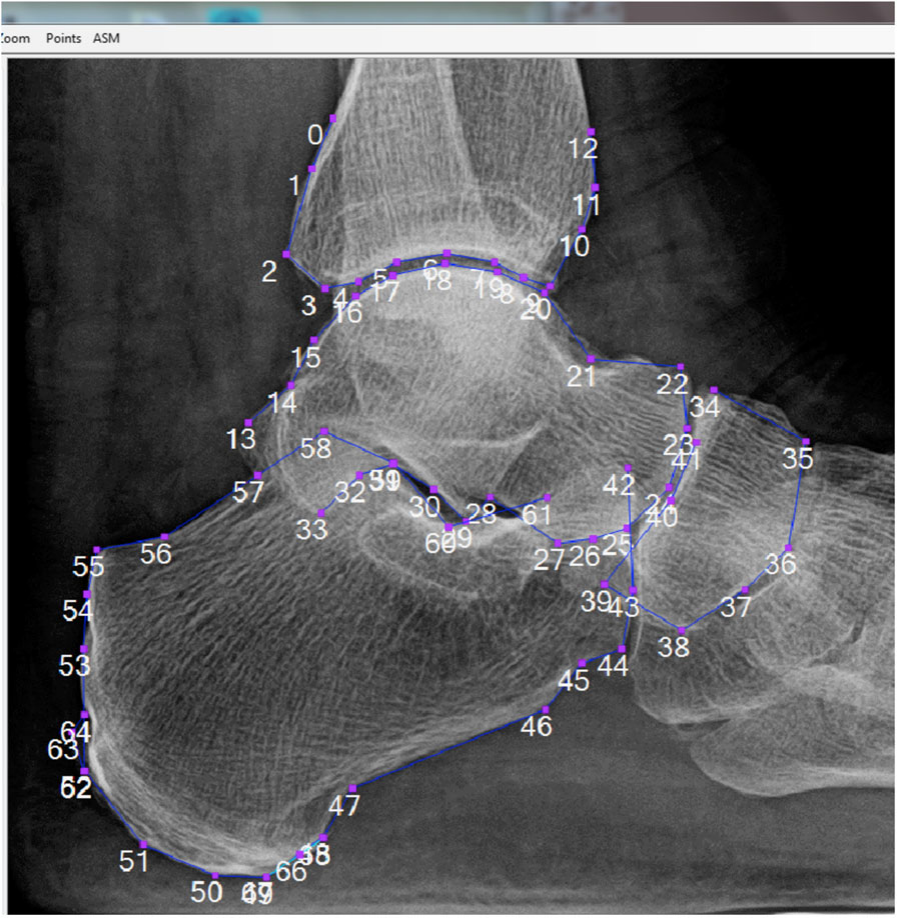
68-point statistical shape model of the lateral ankle

Statistical Shape Modeling (SSM) was developed by Cootes, et al. [10], as a way of segmenting images. The application of SSM as a tool for quantifying bone shape was pioneered by Dr. Gregory [11], to model variations in hip shape which are associated with risk of hip OA [11–13]. For the purposes of this manuscript, “ankle shape” refers to the radiographic two dimensional shapes and relationships (alignment) between shapes, of the distal tibia, talus, calcaneus, and navicular in the lateral view (see also Fig. 1). In the current analysis, we sought to explore the cross-sectional association between ankle shape by SSM and prior report of injury in a large community-based cohort, including African American and white men and women, as a potential intermediary step in the development of OA.

## Methods

This analysis utilizes data from the Johnston County (JoCo) OA Project, a longitudinal community-based cohort study of individuals with and without OA, which has been previously described in detail [14]. Weight bearing radiographs of the ankles and feet were collected only during the third follow-up visit (*n* = 864, 2013–15); data from this time point were therefore used for these cross-sectional analyses. The JoCo OA Project has been continuously approved by the Institutional Review Boards of the University of North Carolina at Chapel Hill (parent project #92–0583, specific for this analysis #14–3273) and the Centers for Disease Control and Prevention (#1820.0).

All participants self-reported age and race. Trained interviewers administered detailed examinations and questionnaires. History of injury was assessed using the question: “Have you EVER injured your right | left ankle badly enough that it limited your ability to walk for at least 2 days?” Symptoms were assessed using the question: “On most days of any one month in the last 12 months did you have pain, aching, or stiffness in any of the following: right | left ankle?”, which was followed by a scale of 0 to 10 representing no to most severe symptoms, dichotomized for these analyses to no symptoms (0) or any symptoms (>0). In a sensitivity analysis, the contribution of physical activity was assessed using a binary variable, “0” if not meeting physical activity guidelines and “1” if meeting this level of physical activity [15].

Lateral views of each ankle were obtained separately with equal weight-bearing on each limb, the foot parallel to the cassette, and the x-ray tube angled at 90 degrees and centered at the base of the first metatarsal (distance = 1 m). The tibiotalar joints were read for Kellgren-Lawrence grade (KLG) by a musculoskeletal radiologist (JBR) using a standardized atlas with good reliability [16]. For this analysis, injury cases (*n* = 108) were all ankles without tibiotalar OA (KLG of 0 or 1) for which participants self-reported prior injury. Non-injured controls were an equal number of randomly selected ankles without tibiotalar OA (KLG of 0 or 1) for which participants did not report a prior injury (according to the question above), for a total sample size of 216.

### Statistical shape modeling (SSM)

The shape of the ankle was determined on the weight-bearing lateral ankle radiographs for all ankles by a trained reader (SL) blinded to other clinical data, by 68 landmark points. The location of these landmark points was detailed in an example image along with descriptive text (e.g. point “18” was to be at the center of the top of the talar dome, see Fig. 1) which was used during training and reading to standardize point placement. Lateral ankle images were selected given the ability to delineate more of the anatomy (distal tibia, calcaneus, talus, and navicular) in comparison to mortise views which show only the tibiotalar joint in detail; no other views were available. Twenty randomly selected films were read by the same reader (SL) or two readers (SL and AEN) one week apart to establish intra- and inter-reader repeatability, respectively, of point placement, as well as intraclass correlation coefficients (ICC) for mode 1.

Once the landmark points were placed on the images, the coordinates were entered into an SSM (*Shape* software, University of Aberdeen), which then used Procrustes and principal components analyses to describe the shape using a series of orthogonal modes of variation [11]. Each mode is a descriptor of a certain amount of variation in the overall shape, and the set of modes (each an independent continuous variable) together describe the overall variation. The scores for modes which together explained 80% of the shape variance (*n* = 19), and for which each mode individually explained at least 1% of the variance, were retained for modeling in order to obtain a parsimonious and stable model.

### Statistical analysis

The 19 mode scores were simultaneously included in a logistic regression model as independent predictors with injury status (history of injury vs. non-injured) as the dependent variable, adjusted for intra-person correlations using the cluster option in Stata [17]. Exploration of further adjustment by sex (referent = female), race (referent = African American), body mass index (BMI), KLG, and symptoms was performed.

## Results

After exclusion of 3 ankles due to obscured landmarks, we evaluated 213 ankles from 194 participants: 71% female, 67% white, with a mean age of 71 years and BMI of 30 kg/m^2^ (Table 1). These 213 comprised 112 right ankles and 101 left ankles. There were 107 injured ankles and 106 non-injured ankles. Of the 19 individuals with both ankles included, 12 were bilateral injured ankles, 4 were mixed injured and non-injured ankles, and 3 were bilateral non-injured ankles. Compared with non-injured, injured ankles were more often from Caucasians (78% vs. 58%) and men (33% vs. 27%), and were more often symptomatic (25% vs. 11%), with no substantial difference by age, BMI, or baseline KLG.

**Table 1:** Sample characteristics overall and by injury status (prior injury vs. non-injured)

	Participants (*n* = 194)	Ankles (*n* = 213)	
	Overall	Prior injury (*n* = 107)	Uninjured (*n* = 106)
Age, mean (SD) years	71.0 (7.8)	70.8 (7.4)	71.2 (8.2)
Body mass index, mean (SD) kg/m^2^	30.3 (5.6)	30.5 (6.5)	30.0 (5.3)
White, n (%)	130 (67.0)	83 (77.6)	61 (57.6)
Female, n (%)	137 (70.6)	72 (67.3)	77 (72.6)
Baseline KLG = 0, n (%)	--	30 (28.0)	40 (37.7)
Symptoms present, n (%)	--	27 (25)	12 (11)

Inter-reader reproducibility was shown as 53% of points placed within 1 mm, 88% within 1.5 mm, and 100% within 2 mm by two independent readers (mean difference 1 mm). For intra-reader repeatability, 63% of points were placed within 1 mm, 90% within 1.5 mm, and 96% within 2 mm by one reader (mean difference < 1 mm). For mode 1, which explained >30% of the total variance for these 20 radiographic shapes, both the inter-reader and intra-reader ICCs were greater than 0.97.

In a model adjusted only for intra-person correlation, statistically significant associations were seen between injury status and modes 1, 6, 13, and 19 (OR^1^, Table 2). In a fully adjusted model (OR^2^, Table 2), only adjustment for race (but none of the other covariates) affected the estimates, such that mode 1 was no longer significant, but estimates for modes 6, 13, and 19 were essentially unchanged. No differences were seen when additional adjustment was made for physical activity (data not shown).

**Table 2:** Associations (Odds Ratio [OR] and 95% confidence interval [CI]) between independent mode of variation scores and injury (injured or non-injured) status

	Shape variance	OR^1^ (95% CI)	OR^2^ (95% CI)
	explained (%)		
Age (per year)	-	-	0.98 (0.93–1.03)
BMI (per unit)	-	-	0.99 (0.93–1.05)
Race	-	-	**3.11 (1.27–7.65)**
Sex	-	-	1.03 (0.48–2.21)
Baseline KLG	-	-	1.04 (0.49–2.18)
Baseline symptoms	-	-	1.80 (0.76–4.24)
Mode 1	16.4	**1.41 (1.03–1.93)**	1.12 (0.77–1.63)
Mode 2	12.3	1.10 (0.80–1.50)	1.07 (0.78–1.47)
Mode 3	7.6	0.84 (0.60–1.16)	0.92 (0.64–1.32)
Mode 4	6.4	1.12 (0.85–1.49)	0.98 (0.72–1.32)
Mode 5	6.1	1.16 (0.82–1.63)	1.16 (0.82–1.64)
Mode 6	5.2	**0.67 (0.49–0.92)**	**0.67 (0.47–0.95)**
Mode 7	3.8	0.99 (0.72–1.37)	0.90 (0.62–1.30)
Mode 8	3.3	1.08 (0.79–1.47)	1.01 (0.73–1.39)
Mode 9	2.8	0.84 (0.62–1.14)	0.79 (0.56–1.09)
Mode 10	2.4	0.89 (0.66–1.19)	0.90 (0.66–1.22)
Mode 11	2.3	0.99 (0.73–1.35)	1.05 (0.73–1.49)
Mode 12	1.8	1.09 (0.79–1.52)	1.16 (0.83–1.63)
Mode 13	1.7	**1.61 (1.17–2.23)**	**1.64 (1.18–2.28)**
Mode 14	1.6	0.83 (0.60–1.13)	0.81 (0.57–1.14)
Mode 15	1.5	1.04 (0.76–1.43)	1.02 (0.74–1.41)
Mode 16	1.4	1.13 (0.80–1.60)	1.21 (0.85–1.71)
Mode 17	1.3	0.86 (0.64–1.15)	0.81 (0.59–1.11)
Mode 18	1.2	1.01 (0.73–1.39)	0.98 (0.71–1.36)
Mode 19	1.1	**0.52 (0.37–0.71)**	**0.54 (0.38–0.75)**

OR^1^: Adjusted only for intra-person correlation

OR^2^: Additionally adjusted for baseline covariates as shown, referent category for race was African American and for sex was female

BOLD = statistically significant result

Given these findings, a separate model with race as the dependent variable was assessed and revealed a strong association between self-reported white race and mode 1 (OR 4.14 [95% CI 2.56–6.71], full data not shown).

**Fig. 2:**
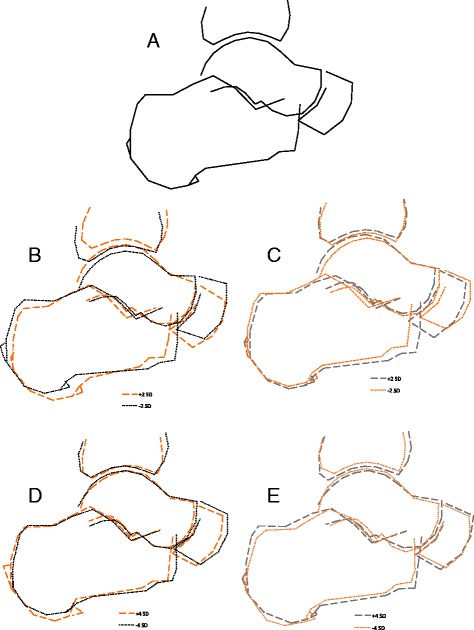
Representation of 2-dimensional modes of lateral ankle shape. Mean shape (**a**) at top. The variation that was associated with injury history is shown in orange, whether it was the positive standard deviation (+SD) change (*long dashes*) or the negative SD (−SD) change (*dotted lines*). For modes 1 (**b**) and 6 (**c**), +/− 2 SD changes are shown, while for modes 13 (**d**) and 19 (**e**), +/− 4 SD changes are shown

Representations of the shape variation for modes 1, 6, 13, and 19 are shown in Fig. 2. For mode 1, a + SD change (associated with injury status and white race) reflects a shift in alignment, of the tibia (more anterior), the navicular (more inferior), and the calcaneus (the latter was also somewhat smaller), with resultant alterations in the tibiotalar, talonavicular, and subtalar joints; the talar dome also appears higher. For mode 6, a –SD change is associated with injury status and reflects a smaller navicular and other minor shifts (e.g. a flatter talar dome and shift in location of the tibiotalar joint). A + SD change in mode 13 was also associated with injury status and visually is primarily related to increased size of posterior calcaneal and plantar enthesophytes. The changes in mode 19 were subtle and difficult to visualize using these methods, although again suggested some shifts in alignment and interbone positioning.

## Discussion

In this community-based cohort, we found that variations in ankle shape are associated with self-reported ankle injury and that there are racial differences in ankle shape. Currently, different modes of SSM can only be qualitatively assessed by visualizing variations in the modes, as there is no standardized reference cohort for joint shape, and thus the variations seen are specific to this population. Most of the variations identified can be summarized as alterations in bone size and in alignment between the bones. In particular, mode 1 demonstrates an apparent shift in relative alignment that affects all 3 (tibiotalar, talonavicular, and subtalar) joints visualized on the lateral ankle radiographs.

Of particular interest, compared to those without injury, a marked change in enthesophytes (mode 13, Fig. 2d) was noted in ankles with prior injury. One could speculate about the direction of this relationship (injury leading to abnormal joint shape or abnormal joint shape increasing the risk for injury), but evidence is not yet available to reveal the nature of this association.

We have previously reported on racial differences in SSM and geometric measures at the hip [13, 18, 19], but no comparable data exists at the ankle, and there are no epidemiologic studies exploring potential effects of race in ankle OA. In work we have recently published, we found that African Americans were somewhat less likely than whites to have radiographic ankle OA (defined as KLG ≥ 2) [3].

While there are no studies of SSM at the ankle, and no other reports of racial differences in ankle OA or ankle shape, there have been attempts to consider bone shape at the ankle joint in OA using other modalities. Using 3-dimensional CT-based models, Schaefer et al., found that mean tibial and talar radii were higher (indicating less curvature), and talar coverage angles were lower, in patients with ankle OA (13 ankles) compared with healthy younger controls. This flatter joint surface could negatively affect the stability and containment of the joint, while the reduced coverage angle suggests less articular support, and could result in a more concentrated load and increased stress on the OA joint [20]. Similarly, Wiewiorski et al. identified higher talar radii, greater sagittal curvature and anterioposterior width of the distal tibia, among patients with primary (non-traumatic) end stage ankle OA (KLG 3–4, 27 patients) compared with healthy controls [21]. The authors postulate that these changes could protect the joint from overuse or reduce pain by reducing range of motion; both of these studies were of patients with existing OA and could not assess changes that might lead to later development of OA. Lee et al. used geometric indices of ankle shape on radiographs to describe alterations in ankle morphology among individuals with lateral malleolar fracture (*n* = 274) and lateral ankle sprain (*n* = 400), finding that the tibia was slightly more anterosuperiorly tilted in the sprain group [22].

Although it is obviously not possible to directly compare our results to these reports, given the differences in methods and populations, there are a few potential commonalities. A + SD in mode 1 includes an anterosuperior tilting of the tibia as noted by Lee et al., for patients following ankle sprain. Also, again only qualitatively, the tibiotalar joint surfaces appear somewhat less congruent in the +2SD variation of mode 1 compared with the -2SD variation, possibly hinting at a less stable joint as seen by Shaefer et al., although further study is needed to confirm these observations.

This study has many strengths, the foremost of which is the large, well-characterized cohort from which the images were drawn, which includes African American and white men and women, allowing consideration of differences by race and sex. Radiographs were obtained in a standard manner by a trained technologist on dedicated equipment, followed by evaluation by a single, reliable, and highly experienced musculoskeletal radiologist. We found good intra- and inter-reader agreement for landmark point placement for this novel 68-point model. We utilized conventional radiography of the ankles in weight-bearing, which is a readily available and clinically relevant modality for assessment of ankle OA.

As with any study, there are also limitations, and we acknowledge that while 2-dimensional radiography is clinically relevant, it does not allow characterization of the 3-dimensional interactions of bones in this complex joint, and further studies using 3-dimensional assessments (incorporating elastic foundation or finite element based modeling [23, 24]) could provide further insight and validation of these preliminary observations. For this initial analysis, we selected and modeled the shape of the ankle from lateral radiographs, but additional information could certainly be obtained from the available mortise views or other specialty views, and differences in positioning could contribute to the observed variation. While radiographs are limited to assessment of bone, this tissue has the highest modulus of elasticity and therefore is likely to drive the level of biomechanical stress in the joint, with subsequent damage to cartilage and other soft tissues. Participants were asked a fairly general question regarding prior ankle injury, so we do not have detailed data about aspects of the injury that may be more or less likely to alter joint mechanics, such as type of injury (e.g., fracture vs. sprain), joint tissues involved, and severity of the injury. Our definition of ankle injury may have captured milder injuries along with those that were more severe, potentially attenuating the observed associations if only severe injuries are related to joint shape. Also, self-report of injury is subject to error due to participants not accurately recalling their injury status, but medical records may not be a reliable source, either, since not every joint injury is reported to a health care professional at the time of the injury. Finally, this analysis is cross-sectional, so we are not able to address causality. That is, did the injury alter the shape (and potentially predispose the ankle to later OA), or was the shape already abnormal and predisposed the ankle to injury (and potentially later OA) in the first place?

This work raises several questions for future study. As part of the continuing JoCo OA Project, we will obtain weight-bearing ankle films in future follow-ups, which will allow us to assess changes in shape that may predispose to incident OA at the ankle as we have done at the hip in prior work. We can also assess changes in ankle shape over time in previously injured versus non-injured ankles. Additionally, these alterations in shape can be analyzed along with other detailed data available from the parent cohort, including biomechanical assessment of static foot structure (high, neutral, or flat arch) and dynamic foot function (over-pronated, neutral, or over-supinated) utilizing plantar pressure measurement data, to better characterize the potential effects of alterations in shape. Future studies could also explore comparisons between predicted joint stresses obtained from 2-dimensional shape models and 3-dimensional methods to see if simpler 2-dimensional models could provide useful clinical information.

## Conclusions

In this community-based cohort, there was an association between variations in ankle shape and prior history of ankle injury, and racial differences in ankle shape were observed (as have been previously noted at the hip). These novel findings may indicate a change in ankle morphology following injury, or that ankle morphology predisposes to injury. In either case ankle shape could be an important factor in the development of ankle OA.

## Abbreviations

BMI: Body Mass Index
CI: Confidence Interval
CT: Computed Tomography
JoCo OA Project: Johnston County Osteoarthritis Project
KLG: Kellgren-Lawrence Grade
OA: osteoarthritis
OR: Odds Ratio
SD: Standard Deviation
SSM: Statistical Shape Modeling
